# Biomimetically Inspired Highly Homogeneous Hydrophilization of Graphene with Poly(l-DOPA): Toward Electroconductive Coatings from Water-Processable Paints

**DOI:** 10.1021/acssuschemeng.2c00226

**Published:** 2022-05-10

**Authors:** Anna Kuziel, Grzegorz Dzido, Rafał G. Jędrysiak, Anna Kolanowska, Bertrand Jóźwiak, Juliette Beunat, Emil Korczeniewski, Monika Zięba, Artur P. Terzyk, Noorhana Yahya, Vijay Kumar Thakur, Krzysztof K. Koziol, Sławomir Boncel

**Affiliations:** † Department of Organic Chemistry, Bioorganic Chemistry and Biotechnology, Silesian University of Technology, Krzywoustego 4, 44-100 Gliwice, Poland; ‡ Enhanced Composites and Structures Centre, School of Aerospace, Transport and Manufacturing, Cranfield University, Cranfield, MK43 0AL Bedfordshire, U.K.; § Department of Chemical Engineering and Process Design, Silesian University of Technology, Strzody 7, 44-100 Gliwice, Poland; ∥ Cambridge Graphene Centre, Engineering Department, University of Cambridge, 9 JJ Thomson Avenue, CB3 0FA Cambridge, U.K.; ⊥ Faculty of Chemistry, Physicochemistry of Carbon Materials Research Group, Nicolaus Copernicus University in Toruń, Gagarin Street 7, 87-100 Toruń, Poland; # Department of Fundamental and Applied Sciences, Universiti Teknologi Petronas, 32610 Seri Iskandar, Perak Darul Ridzuan, Malaysia; ¶ Spin Eight Nanotechnologies Sdn. Bhd. 28, Persiaran Jelapang Maju 7, Kawasan Perindustrian Ringan Jelapang Maju, 30020 Ipoh, Malaysia; ∇ Biorefining and Advanced Materials Research Center, SRUC, EH9 3JG Edinburgh, U.K.; ○ School of Engineering, University of Petroleum & Energy Studies (UPES), 248007 Dehradun, India

**Keywords:** graphene, covalent functionalization, water-processing, poly(l-DOPA), hydrophilization, electroconductive coatings

## Abstract

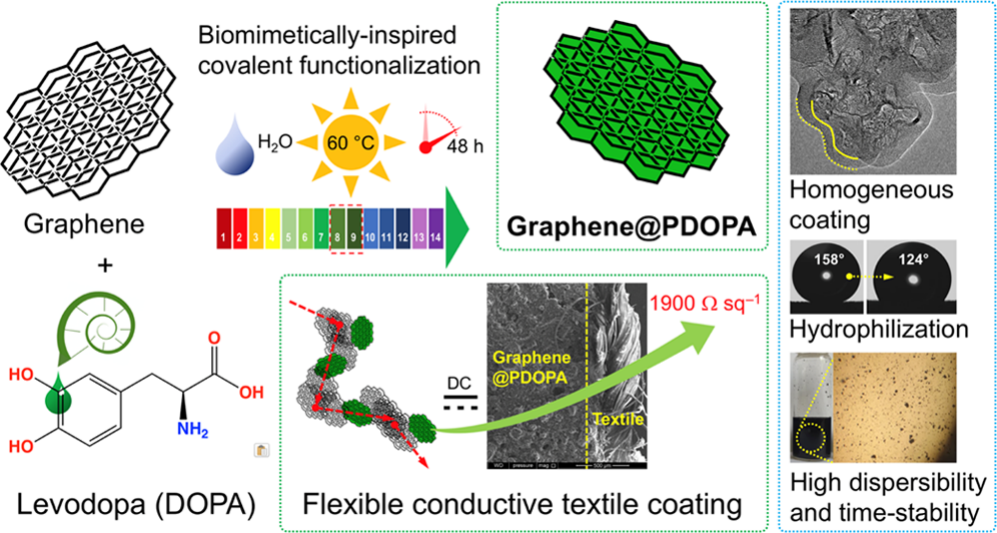

Water-based processing of graphene—typically considered as physicochemically incompatible with water in the macroscale—emerges as the key challenge among the central postulates of green nanotechnology. These problematic concerns are derived from the complex nature of graphene in the family of sp^2^-carbon nanoallotropes. Indeed, nanomaterials hidden under the common “graphene” signboard are very rich in morphological and physicochemical variants. In this work, inspired by the adhesion chemistry of mussel biomaterials, we have synthesized novel, water-processable graphene–polylevodopa (PDOPA) hybrids. Graphene and PDOPA were covalently amalgamated via the “growth-from” polymerization of l-DOPA (l-3,4-dihydroxyphenylalanine) monomer in air, yielding homogeneously PDOPA-coated (23 wt %) (of thickness 10–20 nm) hydrophilic flakes. The hybrids formed >1 year stable and water-processable aqueous dispersions and further conveniently processable paints of viscosity 0.4 Pa·s at 20 s^–1^ and a low yield stress τ_0_ up to 0.12 Pa, hence exhibiting long shelf-life stability and lacking sagging after application. Demonstrating their applicability, we have found them as surfactant-like nanoparticles stabilizing the larger, pristine graphene agglomerates in water in the optimized graphene/graphene–PDOPA weight ratio of 9:1. These characteristics enabled the manufacture of conveniently paintable coatings of low surface resistivity of 1.9 kΩ sq^–1^ (0.21 Ω·m) which, in turn, emerge as potentially applicable in textronics, radar-absorbing materials, or electromagnetic interference shielding.

## Abstract 2

Water-based processing of graphene by means of a natural polymer polylevodopa toward stable and functional electroconductive coatings for textronics was elaborated and cross-verified.

## Introduction

Water-based processing of graphene—the most studied 2D sp^2^-carbon allotrope^1^—conforms the principles of green chemistry.^2^ Such technology is vital as graphene nowadays represents the nanomaterial of the most rapidly growing interest from the world of both science and industry.^3^ This fact arises from its excellent thermal,^4,5^ electrical,^6^ optical,^7^ and mechanical^8^ properties, though reserved for the individualized nanoflakes at the nanoscale.^9^ What is crucial from the processing point of view, graphene demonstrates strong van der Waals inter-flake interactions, and hydrophobicity governed by the lateral size—smaller graphene flakes can be considered as overall amphiphilic^10−12^ with the hydrophilic edges^11^—and, extrinsically, by adsorption of airborne hydrocarbons (graphene and graphite are in fact slightly hydrophilic).^13,14^ The amalgamation of graphene with various matrices (polymers, metals, and ceramics), and hence its physicochemical compatibilization, can lead to numerous scalable applications. Unfortunately, dispersion of graphene is challenging due to the strong intersheet van der Waals attractions (via π–π stacking) causing aggregation and/or precipitation.^15^

A variety of organic solvents inducing repulsive forces after interflake penetration and on-flake adsorption such as benzyl benzoate, *N*-methyl-2-pyrrolidone, and dimethyl sulfoxide could be applied as graphite exfoliating agents and graphene dispersants, respectively.^16^ However, the most pressing technological requirements for the dispersing media are volatility and zero/minimal toxicity, as for water or ethanol.^17^ The latter green dispersants, as the key components in the more complex systems such as elastomers, could lead to more convenient and safer processability toward flexible electroconductive coatings^18^ for textronics,^19,20^ radar-absorbing materials (RAMs),^21,22^ or electromagnetic interference (EMI) shielding.^23^

To enhance the water dispersibility of graphene (understood in the broadest sense according to the ISO standardization ISO/TS 80004-13:2017) and to improve the performance of the graphene-based nanocomposites, several physicochemical modifications have been applied. These structural adaptations involve, inter alia, treatment of carboxylic acid groups retained on the chemically reduced graphene oxide (rGO), grafting of hydrophilic groups (epoxy, hydroxyl, amine, and carboxylic) on graphene oxide (GO) through chemical reactions,^24−26^ or physical adsorption of stabilizers such as surfactants,^27−29^ polymers,^30−32^ or small molecules.^33−35^ Consequently, rather than using high crystallographic quality and pristine graphene—the availability of which is limited to small quantities due to the troublesome large-scale production (via mechanical^36^ or chemical exfoliation^37,38^ and chemical vapor deposition^40^)—GO or rGO emerged thus far as the materials of choice.^10,39^ However, GO flakes which have no clearly defined chemical structure are not only difficult to process^40^ but also exhibit the yet unraveled and complex cyto- and ecotoxicity profiles^41,42^ and undergo fragmentation in the aqueous environments.^43^ However, predominantly, GO does not retain the fundamental properties of graphene, with mechanical and electrical as the key ones.^44,45^ In turn, scalable water-based dispersions, inks, or paints/pastes can be prepared under a rather intense mechanical assistance from (bath to probe) ultrasonication to microfluidization and have led so far to the functional solutions mainly in the area of textronics. Nevertheless, they still require a pretreatment with ethylcellulose,^46^ a mixture of sodium deoxycholate and carboxymethylcellulose sodium salt,^47^ sodium silicate,^48^ sodium salt of flavin mononucleotide,^49^ or poly(*N*-vinyl pyrrolidone)^50^ (ultrasonication). Proteins can also serve as biofriendly surfactants, while adhesion is the key prerequisite for the stabilization of nanoparticles in water. It is well known that mussels strongly adhere to wood/stones under high shear stress conditions from the water flows. This behavior stems from the mussel foot functional proteins containing high contents of two amino acids: l-3,4-dihydroxy phenylalanine (levodopa, l-DOPA) and lysine.^51^ Such characteristics have instantly paved the way for the mimicking of sustainable adhesives and stabilizers toward the new applications in materials science (including, e.g., wastewater treatment^52^), biology, and biomedicine.^53^

In the pursuit of developing novel physicochemically modified graphene of enhanced hydrophilicity and simple but green processing, we hypothesized its covalent functionalization with l-DOPA (precursor of dopamine) as the particularly promising one. l-DOPA, being an in vivo instantly available catecholamine, oxidizes to polylevodopa (PDOPA). This reaction, proceeding under only the mechanical stirring regime, at the solid–liquid interface might form a controllable layer of excellent adhesiveness/affinity to numerous materials.^54−57^ Such a biologically inspired functionalization, essential in the formation of mussels, could be “translated” into the surface modification of graphene with l-DOPA and the subsequent interface polymerization via the “grafting-from” mechanism. This route could be effective in preventing graphene from the excessive agglomeration in the polar solvents.

Taking all the above into account, we have successfully performed the functionalization of graphene toward its individualized, hydrophilic, and thus water-processable nanoparticles. The resulting material displays the enhanced stability of dispersions among variously functionalized graphene flakes. The optimized hybridization with the pristine graphene flakes, serving as the active components of acrylic paints, led to the flexible, on-textile electroconductive coatings of excellent electrical characteristics yet not achieved by the other available green approaches.

## Materials and Methods

### Materials

Graphene CamGraph G3 was supplied by Cambridge Nanosystems Ltd., UK. l-DOPA (≥98%) and Tris-base (tris(hydroxymethyl)aminomethane, 2-amino-2-(hydroxymethyl)-propane-1,3-diol) (≥99.8%) were purchased from Sigma-Aldrich. All of the solutions and dispersions were prepared using distilled water (0.5 μS cm^–1^). Transparent aqueous base SX 150 acrylic (dedicated to screen printing), used for the preparation of electroconductive paints, was purchased from Sico Screen Inks NV (Merchtem, Poland).

### Methods

#### Functionalization of Graphene and Preparation of Graphene Dispersions

Graphene (G3, 1.00 g) and l-DOPA (2.00 g) were dispersed/dissolved in the solution of Tris-base in water (10 mM) (pH = 8.5, 1.0 L). The reaction mixture was placed in a round-bottom flask (2 L) equipped with a reflux condenser and a mechanical stirrer and sealed.^53^ After that, the polymerization reaction was initiated thermally and continued for 48 h at 60 °C using a heating mantle. Upon processing, the dispersion changed color from yellow to brown. The postreaction mixture was centrifuged (10,000 rpm, 30 min), and the resulting black powder (1.20 g),^58^ that is, poly(l-DOPA)-functionalized graphene (G3@PDOPA), was washed with water (1.0 L) and dried at 80 °C for 48 h. Exactly the same procedure was applied for the blank experiments, that is, the samples composed only from l-DOPA or only from graphene G3 flakes.

To evaluate the hydrophilic/hydrophobic behavior of graphene and G3@PDOPA, the corresponding aqueous dispersions (2 mg mL^–1^) were prepared. After weighing out the appropriate amounts of graphene and water, the mixtures were ultrasonicated (Bandelin Sonorex RK 106, 35 kHz, rated power of 480 W) for 10 min. Additionally, in order to evaluate the dispersion effect of l-DOPA operating via the optional noncovalent modifications, we also have prepared the graphene dispersion but disabling polymerization of l-DOPA.

#### Preparation of Electroconductive Paints and Painting

Pristine graphene G3 and G3@PDOPA were used for the preparation of electroconductive paints. Deionized water (70 mL) and graphene (1.00 g) were ultrasonicated for 15 min. Next, the dispersions were homogenized (Bosch mixer CNSM13, 800 W) for 15 min in SX-150 [ecofriendly, water-dilutable transparent screen-printing base containing no formaldehyde and poly(vinyl chloride)] to obtain the final graphene nanoparticle concentration of 10 wt % per base.^59^ Subsequently, the paints were manually applied on the cotton textile using a paintbrush (size, 24; natural bristles; path, 20 mm; 15 × 5 cm paths of the target thickness ca. 100 μm). The coating thickness was monitored during the painting and determined using an Electronica Universal Micrometer LINEAR 0–25 mm/0.001 mm thickness gauge (Dunstable, UK). Similarly, paints containing G3@PDOPA and the mixture of pristine graphene G3 and G3@PDOPA in various ratios (9:1, 8:2, and 1:1) were prepared.

#### Characterization

Scanning electron microscopy (SEM), high-resolution transmission electron microscopy (HR-TEM), Raman spectroscopy, Fourier transform infrared spectroscopy (FTIR), ^1^H nuclear magnetic resonance (^1^H NMR), electrospray ionization mass spectrometry (ESI-MS), and thermogravimetric analysis (TGA) were performed to characterize the changes in morphology and (surface) physicochemistry of G3 flakes, PDOPA, and G3@PDOPA. The SEM images were taken on a SIX HITACHI S-3400 N SEM system (Hemer, Germany). Nanomorphology of the graphene nanofillers was observed using HR-TEM with a TEM F20X-TWIN system (FEI-Tecnai) (Hillsboro, OR, USA) operated at 200 kV. One drop of a sample dispersion in ethanol (96%, p.a.) was placed on a copper grid coated with an ultrathin amorphous carbon film and then dried under ambient conditions. Raman spectra were obtained using (Renishaw, Germany) a green laser (532 nm) focused on a sample using 20× optical zoom, with the laser power of 5% and the exposure time of 10 s. For each material, three accumulations were collected in three locations of the sample. A Thermo Scientific FTIR Nicolet 6700 spectrometer was used for the IR spectroscopic studies of the samples. ESI-MS spectra were acquired on an ESI/MS spectrometer 4000 QTRAP LC/MS/MS system (AB SCIEX) in the positive and negative modes of ionization from the samples dissolved/dispersed in methanol. Combustion elemental analyses were obtained using a PerkinElmer 240C apparatus. TGA curves were recorded using a PerkinElmer TGA 8000 thermobalance in a heating rate of 10 °C min^–1^ under an argon atmosphere. Additionally, particle size distribution (Malvern Zetasizer Nano S90) and zeta potential (Malvern Zetasizer Nano Z) analyses were performed. The microstructure of dispersions was characterized using an optical microscope Levenhuk 870t. The electric resistance (Ω) of the paths was measured using the voltage method in a custom-designed voltage measurement system.^59^ Due to the possibly heterogeneous nature of the electroconductive coatings, the system was customized to textiles with the extended contact surface of the path/electrode. This approach enabled us to reduce the heterogeneity of the electric field and the distribution of the current density.^59^ The measurements were done by placing multimeter aluminum electrode block probes, with the distance between them of 10 cm, onto the surface of coating. We have measured the resistance for each of the coatings in triplicate.

The content of surface oxygen groups in all samples was determined by Boehm titration,^60^ and the procedure was as follows: functionalized (and pristine, i.e., blank sample) graphene (60 mg) was dispersed for 48 h under continuous magnetic stirring in the specified aqueous reaction base solutions: (1) 0.01 M NaOH, 60 mL; (2) 0.01 M Na_2_CO_3_, 30 mL; and (3) 0.01 M NaHCO_3_, 60 mL. The mixture was filtered using a 0.2 mm PTFE filter and then 10 mL (or 5 mL in the case of Na_2_CO_3_) of the appropriate filtrates was titrated with 0.01 M HCl_aq_ (20 mL). The pH of the final solutions was measured using an EcoTestr2 pH meter. Each titration was performed in triplicate. As blank samples, 10 mL of each reaction base (5 mL in the case of Na_2_CO_3_) was used. In turn, to determine the content of amine group,^61^ 20 mg of each sample was added to 10 mL of HCl_aq_ (0.05 M). The mixture was shaken for 30 min in a sealed container under a N_2_ atmosphere and then filtered to remove graphene from the solution. Immediately after the filtration, the solution was titrated with NaOH (0.05 M). The pH of the final solutions was measured using the abovementioned pH meter.

The water contact angle (WCA) (five measurements, standard deviation < 5%) was measured at 25 °C (relative humidity, 35%) using the goniometric system used by us previously.^62^ The samples of G3, G3@PDOPA, and PDOPA were sprayed on the surface of polished silicone (1 × 1 cm, Institute of Electronic Materials Technology, Lukasiewicz Research Network, Warsaw, Poland) using air under a pressure of 2.5 bar airbrush (Fine-Art FA-180X, with a 0.2 mm nozzle). The dispersions/solutions were sprayed up to a coverage of 0.1 mg cm^–2^. For G3 and G3@PDOPA, the suspensions (1.0 mg mL^–1^) in isopropanol (99.7%, pure p.a., Chempur, Piekary Slaskie, Poland) were used after sonication (10 mL, 20 W, 40 s, SONOPULS HD 4200). For the PDOPA sample, 20% MeOH_aq_ (pure p.a., Chempur, Piekary Slaskie, Poland) addition was required to stabilize the suspension (but still 10 mL of 1 mg mL^–1^ suspension was prepared). Spray-coated silicone samples were air-dried at room temperature for 24 h to evaporate the remaining solvents.

The rheological properties of paints were characterized at 40 °C using a Brookfield LVDVII+Pro spring-type rotational viscosimeter with DIN-87 spindles. We conducted shear rate ramp-up (1–50 rpm, 1.29–64.5 s^–1^) and ramp-down (50–1 rpm, 64.5–1.29 s^–1^) tests with 30 s intervals. Also, we experimentally determined the yield stress of the samples by subjecting them to very low shear (1 rpm, 1.29 s^–1^) and then stopping the rheometer drive. The deformed spring caused the rotating cylinder to move back, reducing the torque and shear stress on the surface until the cylinder stopped. When it happened, the value of the shear stress indicated by the viscometer was precisely the yield stress of paints.^63^ The uncertainty of rheological measurements was ±5.4%.

## Results and Discussion

### Synthesis and Characterization of Pristine and Functionalized Graphene

**Figure 1:**
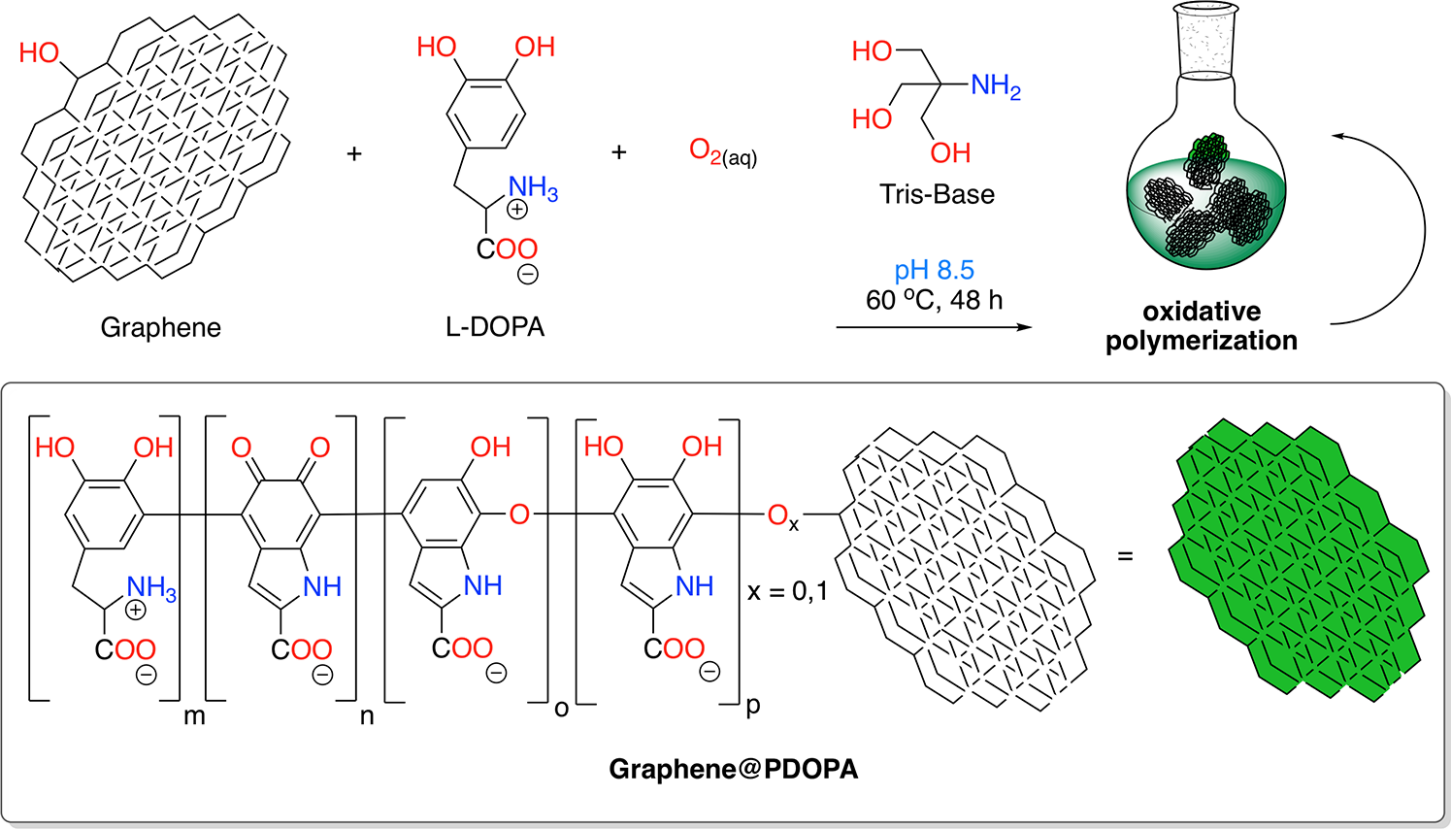
Scheme of synthesis of G3@PDOPA.

According to the TEM measurements, G3 flakes had thickness of 1–5 nm (representing 3–15 layers). The in-plane dimension of the G3 graphene flakes (the flake diameter) was in the range of 400 ± 150 nm, as determined by SEM, and the Brunauer–Emmett–Teller (BET) surface area equaled to 130 ± 5 m^2^ g^–1^ (measured according to the BET theory of bulk samples).^11^ The synthesis of G3@PDOPA was performed via the complex “growth-from” polymerization mode (Figure 1).^64^

Both aromatic sp^2^- and sp^3^-carbon as well as oxygen atoms from hydroxyl groups (referring to the further analyses)—shown in Figure 1 as (O)_*x*_ in the G3@PDOPA structure, where *x* = 0 and 1—could serve as the reactive hot spots.^65^ These centers could capture the l-DOPA radicals generated from the oxidation of monomer molecules by dissolved air. This behavior was confirmed in the polymerization of catechol (1,2-benzenediol) moieties in the presence of metallic surfaces such as silver^66^ or surface hydroxyl groups from iron oxides.^67^ This reaction would yield covalently modified graphene, excessively coated with PDOPA negatively charged in water by carboxylates and phenolates. Overall, these prerequisites would correspond to the covalent nature of the herein graphene functionalization. Particularly, C-sp^3^ defects, also confirmed by complementary analyses, would be the reactive centers in the free-radical initiation of air-induced polymerization of l-DOPA as, in fact, they would exhibit benzylic-type (Ph_3_CH → Ph_3_C^•^) reactivity.^68,69^

**Figure 2:**
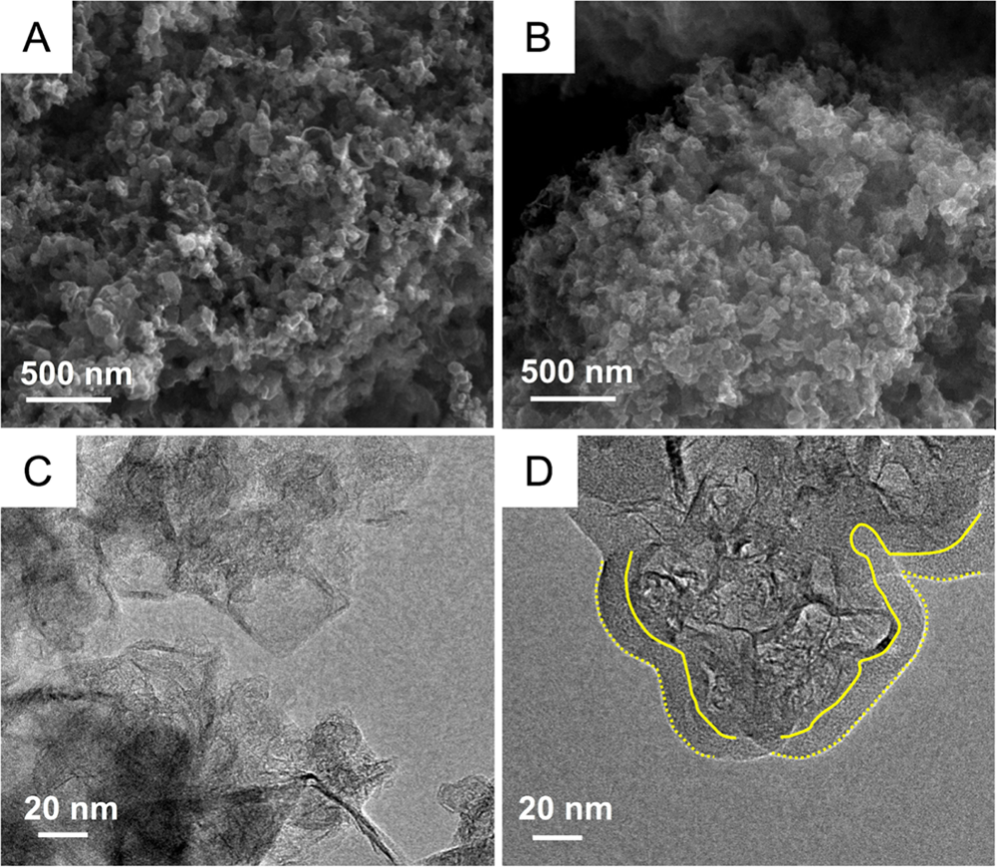
SEM images of (A) G3 graphene and (B) G3@PDOPA, and TEM images of (C) G3 graphene and (D) G3@PDOPA; the solid and dotted lines in (D) correspond to graphene/PDOPA and DPOPA/air interfaces, respectively.

The morphology of the functionalized graphene flakes has been evaluated using SEM and TEM (Figure 2). The imaging revealed that pristine flakes emerged as highly corrugated overlaying few-layer graphene flakes arranged into a characteristic crumpled-like structure produced in the absence of a substrate or exfoliation medium (A) of irregular edges (C). After functionalization with PDOPA, the modified grains emerged slightly thicker, as visible with the unarmed eye at the same magnification (B). Importantly, the PDOPA layers, 10–20 nm in thickness, appeared to homogeneously wrap the graphene flakes (D).

Such a molecular hybridization—associated with the π–π stacking between the few-layer graphene flakes and PDOPA benzene/1*H*-indole rings as well as the π–cation interactions between the protonated amine group (−NH_3_^+^) from the PDOPA zwitterionic blocks and π–graphene layer—could benefit from the steric stability in the aqueous environments. On the other hand, van der Waals and π–π stacking interactions between the graphene flakes—particularly at the highly developed surface area of intrinsically hydrophobic flakes—might yield a substantial agglomeration over time. This phenomenon makes graphene a hard-to-process material to such an extent that it is practically impossible to disperse graphene sheets in water without the assistance of dispersing agents. However, when placed in an oil/water mixture, graphene becomes wettable and actually exhibits amphiphilic properties driven by the hydrophilic edge domains.^11^

**Figure 3:**
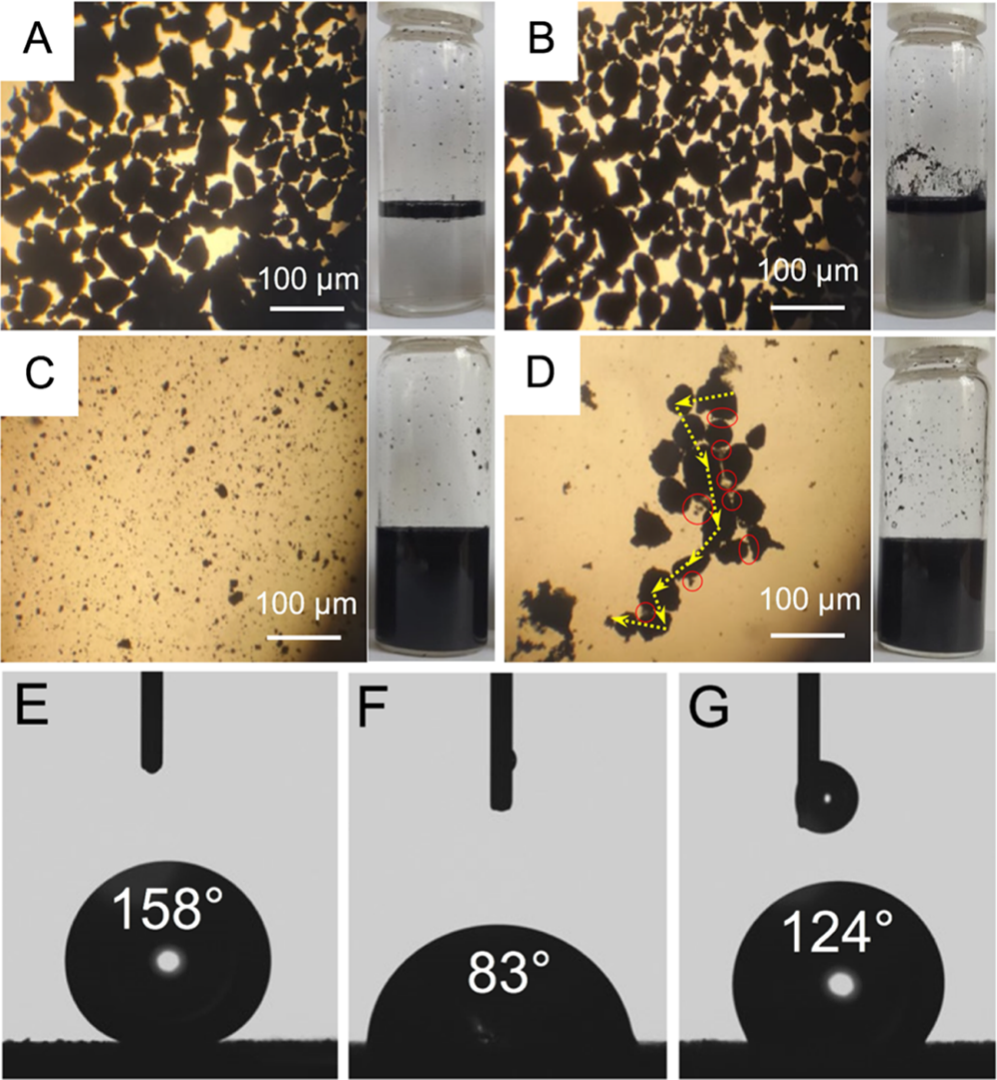
(A) Micrograph of pristine graphene in water; (B) micrograph of pristine graphene with l-DOPA in water; (C) micrograph of functionalized graphene G3@PDOPA in water; (D) micrograph of G3@PDOPA and pristine graphene in 1:9 ratio (w/w); the arrows and circles/ellipses in (D) show interconnects potentially leading to form percolation thresholds and stabilizing effect of smaller, individualized G3@PDOPA particles; insets in (A–D): photographs of the vials containing the corresponding aqueous dispersions. The images showing WCA on graphene G3 (E), PDOPA (F), and G3@PDOPA (G).

To observe the macroscopic behavior of graphene and G3@PDOPA, we have studied their: (a) behavior in water, with the support of optical microscopy and (b) wettability (Figure 3). Pristine graphene flakes agglomerated in water (Figure 3A) up to a few hundred micrometers, and only slightly smaller flakes could be formed after the addition of l-DOPA monomer (Figure 3B). The noncovalent treatment of graphene did not bring any significant improvement in dispersibility as compared to G3@PDOPA (Figure 3C). After functionalization with PDOPA, graphene formed fine, >1 year stable dispersions in water. When we examined the dispersion containing pristine graphene G3 and G3@PDOPA (ratio 9:1), it became clear that the strong interactions between G3 and G3@PDOPA could provide linkages between the flakes, similarly making the dispersion even more stable (Figure 3D).

Figure 3E–G shows the values of WCA on the studied samples. For the unmodified graphene G3—the most hydrophobic material—WCA (158°) was found larger than those determined by other authors for different graphene samples.^70^ Indeed, G3 does not contain a significant amount of oxygen functionalities, but one could also expect a perpendicular orientation of some flakes, causing a strong Cassie–Baxter effect, further increasing the WCA value. On the other hand, the abundance of oxygen-containing groups makes the PDOPA surface hydrophilic—the WCA was found equal to 83°. The surface of the target G3@PDOPA hybrid showed almost the average WCA value between G3 and PDOPA, that is, 123°. Overall, the hydrophilicity of graphene was increased due to, from one side, strong π–π interactions of the aromatic PDOPA rings (tail-like) and the graphene flakes,^58,71−73^ while from the other side, the high content of the −COOH, −OH, and −NH_2_ serving as polar heads oriented outward water. Such a superstructure would hence promote dispersibility and wettability of graphene in/by water.

As a technique complementary to optical microscopy, dynamic light scattering (DLS) was performed to obtain the lateral size distribution of the water-dispersed flakes, which actually confirmed the above-observed tendencies. Table 1 shows the hydrodynamic diameter of graphene–PDOPA and the neat PDOPA particles dispersed in water (see also Supporting Information, Figure S1). The *z*-average particle diameter corresponds to the intensity-weighted mean hydrodynamic size of the investigated particles. The *z*-average values in Table 1 are the arithmetic mean of five independent measurement series for which the polydispersity index was 0.35 (PDOPA) and 0.32 (G3@PDOPA), respectively, which is in the acceptable range for this method.

**Table 1:** Particle Size and Zeta Potential Determined by DLS for PDOPA and G3@PDOPA Aqueous Dispersions

DLS parameter	PDOPA	G3@PDOPA
*z*-average particle diameter, nm	223 ± 3	342 ± 19
Zeta-potential ζ, mV	–31.9 ± 0.8	–25.4 ± 1.6

The agglomerate sizes derived from the DLS data can be complex due to a number of shape modes of the graphene nanoparticles. Commonly, DLS is reserved for the analysis of spherical particles.^74^ However, measuring the hydrodynamic diameter of graphene flake agglomerates can be done by DLS,^75^ though one must consider that DLS rather shows only the size distribution of different particles. Accordingly, the hydrodynamic diameter of G3@PDOPA particles was reduced up to 342 nm as compared to the immeasurably large pristine graphene flakes in the suspension. In turn, zeta-potential measurement is one of the useful techniques to determine the stability of the carbon nanomaterials in the solvents, as the optical observation could not determine the absolute stability of dispersions. Again, the zeta-potential of pristine graphene G3 could not be recorded due to the strong hydrophobic character and its immiscibility/nonwettability with/by water. The stability of G3@PDOPA dispersion could be directly correlated to high negative ζ-values (−25.4 ± 1.52 mV). For PDOPA solution, the ζ-values are even more negative (−31.9 ± 0.79 mV) that corresponds to even better stability of the PDOPA dispersion (Table 1). GO, in contrary to G3@PDOPA, could be characterized even with the more negative value of ζ-potential, that is, less than −60 mV—due to the larger content of dissociating oxygen-containing groups.^76,77^

**Figure 4:**
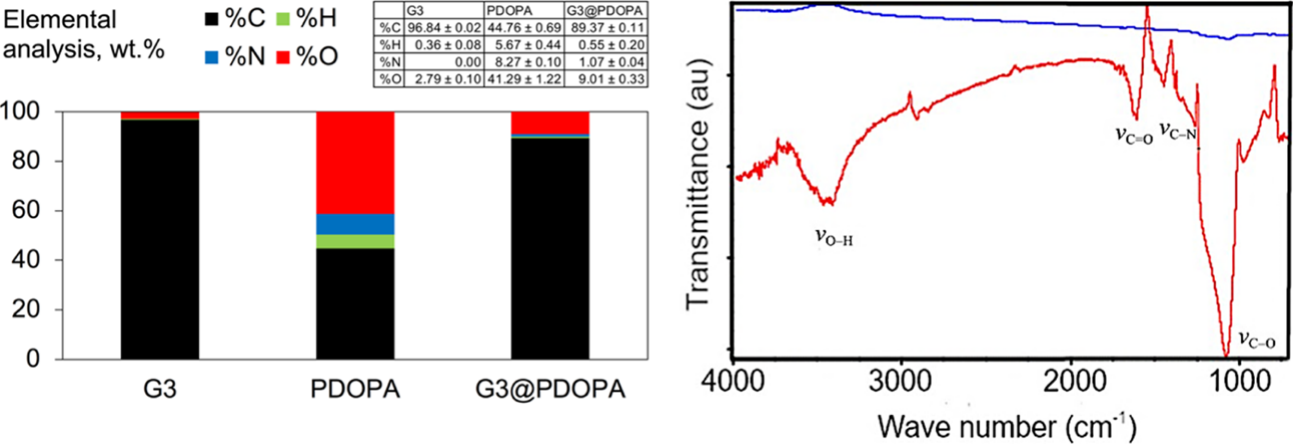
Combustion elemental analysis (left) and FTIR spectra (right) of G3@PDOPA (red) vs G3 (blue).

To further study the nature of graphene functionalization with PDOPA, we have performed the combustion elemental (C, H, and N) analysis (EA) (Figure 4, left) (the oxygen content wt % was determined indirectly by subtracting the CHN content from 100%) and acquired FTIR spectra (Figure 4, right) of G3@PDOPA in the background of its synthetic precursors.

Pristine graphene (ca. C_46_H_2_O) was composed of 97 wt % of carbon and only a small number of oxygen groups accompanied by adsorbed water. In the case of G3@PDOPA, the oxygen and nitrogen content increased to ca. 9 and 1%, respectively, clearly revealing the presence of PDOPA. The more qualitative approach, that is, Boehm titration, has further verified these results (Table 2).

**Table 2:** Content of the Oxygen Groups as Determined by Boehm Titration

oxygen functionality, conc.	G3	PDOPA	G3@PDOPA
*n*_COOH_, mmol g^–1^	0.90 ± 0.20	2.10 ± 0.35	1.10 ± 0.31
*n*_OH (PhOH-like)_, mmol g^–1^	1.14 ± 0.25	2.50 ± 0.32	1.75 ± 0.42
*n*_lactone_, mmol g^–1^	0.00 ± 0.15	0.60 ± 0.29	0.49 ± 0.30

Pristine graphene had a small amount of oxygen groups on the surface—carboxylic (0.90 mmol g^–1^) and phenol-like groups (1.14 mmol g^–1^)––which correspond to the minimum of 3.6 wt % of oxygen. After functionalization with PDOPA, the content of carboxylic groups in G3@PDOPA increased to 1.10 mmol g^–1^, phenol-like groups to 1.75 mmol g^–1^, and lactone to 0.49 mmol g^–1^. These characteristics emerge with the grafting of graphene with the complex and cross-linked poly(l-DOPA). Additionally, during the l-DOPA polymerization, a fraction of carboxylic groups could undergo decarboxylation, while some hydroxyl groups oxidize to quinone-like moieties (refer back to Figure 1).^78^ In parallel, titration revealed that pristine graphene contained no amino groups, while after functionalization, the content of amine groups increased to 3.20 ± 0.15 mmol g^–1^. Comparing the normalized FTIR spectra of graphene versus G3@PDOPA, one can immediately notice rather striking differences in the absorption band locations and their intensities. The strong peaks around 3430 cm^–1^ (ν_O–H_) and 1100 cm^–1^ (ν_C–O_), absent in pristine graphene, resulted from the −OH stretching vibration from PDOPA. In addition, the intense peak at 1630 cm^–1^ could be ascribed to the stretching vibration of the carbonyl group (ν_C=O_). Further, weaker signals at ca. 3000 cm^–1^ correspond to the stretching vibrations of C_Ar_–H (ν_C–H_). The N–H bending as well as the C–N stretching vibrations resulting from the presence of amine groups can be observed at 1520 (δ_N–H_) and 1270 cm^–1^ (ν_C–N_), respectively. Additionally, the ^1^H NMR spectra of PDOPA and G3@PDOPA confirmed: (1) a more pronounced oxidation level of phenolic hydroxyl to 1,2-keto groups as signals at 8.10 and 8.25 ppm were significantly reduced in G3@PDOPA and (2) a higher level of cross-linking as all of the ^1^H NMR signals were significantly broadened (Supporting Information, Figure S2). The presence of l-DOPA units in the oligo- and polymers (linear and cross-linked) in PDOPA and G3@PDOPA was further confirmed via ESI-MS with an exemplary identification of the partially oxidized l-DOPA pentamer and dimer (Supporting Information, Figures S3–S6).

**Figure 5:**
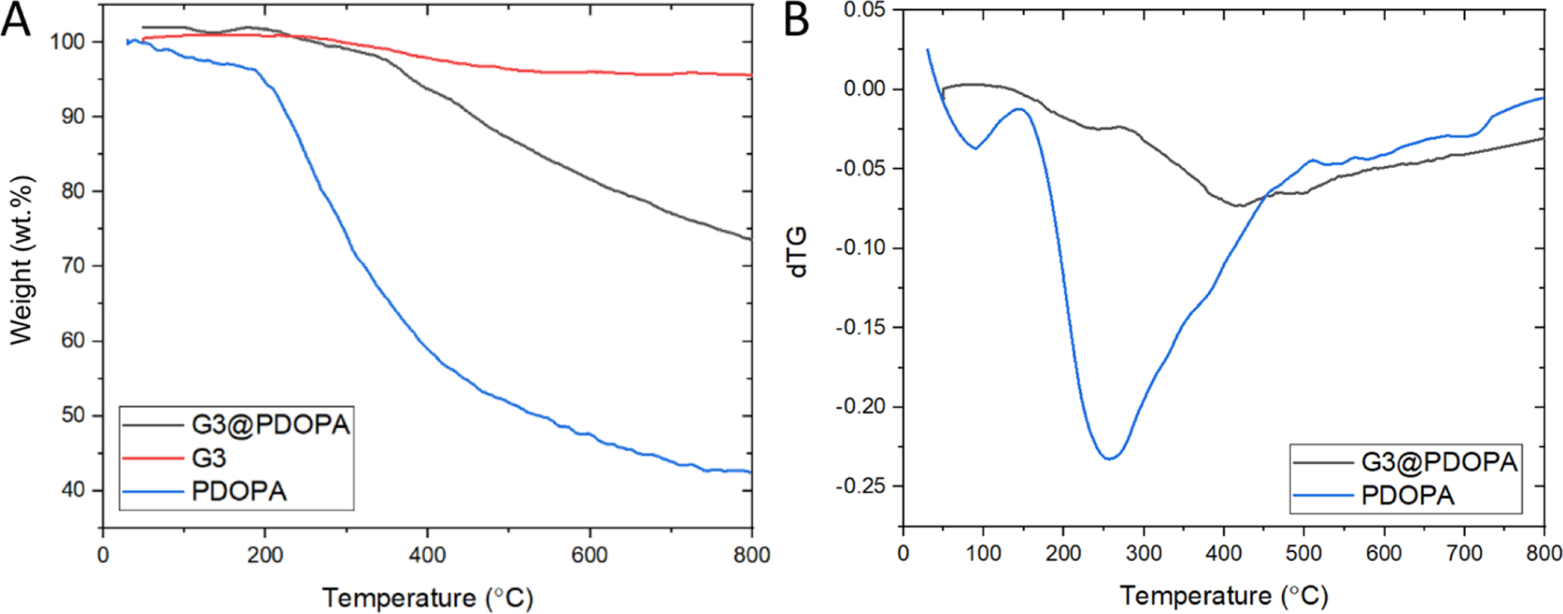
(A) TGA curves of PDOPA, graphene G3, and G3@PDOPA; (B) dTG curves of PDOPA and G3@PDOPA.

In order to investigate the thermal stability of the samples and to determine the PDOPA content in the functionalized graphene, TGA was carried out in an argon atmosphere, that is, under pyrolytic conditions. Figure 5 shows the corresponding TGA (A) and dTG (B) curves of pristine graphene G3, neat PDOPA, and G3@PDOPA.

**Figure 6:**
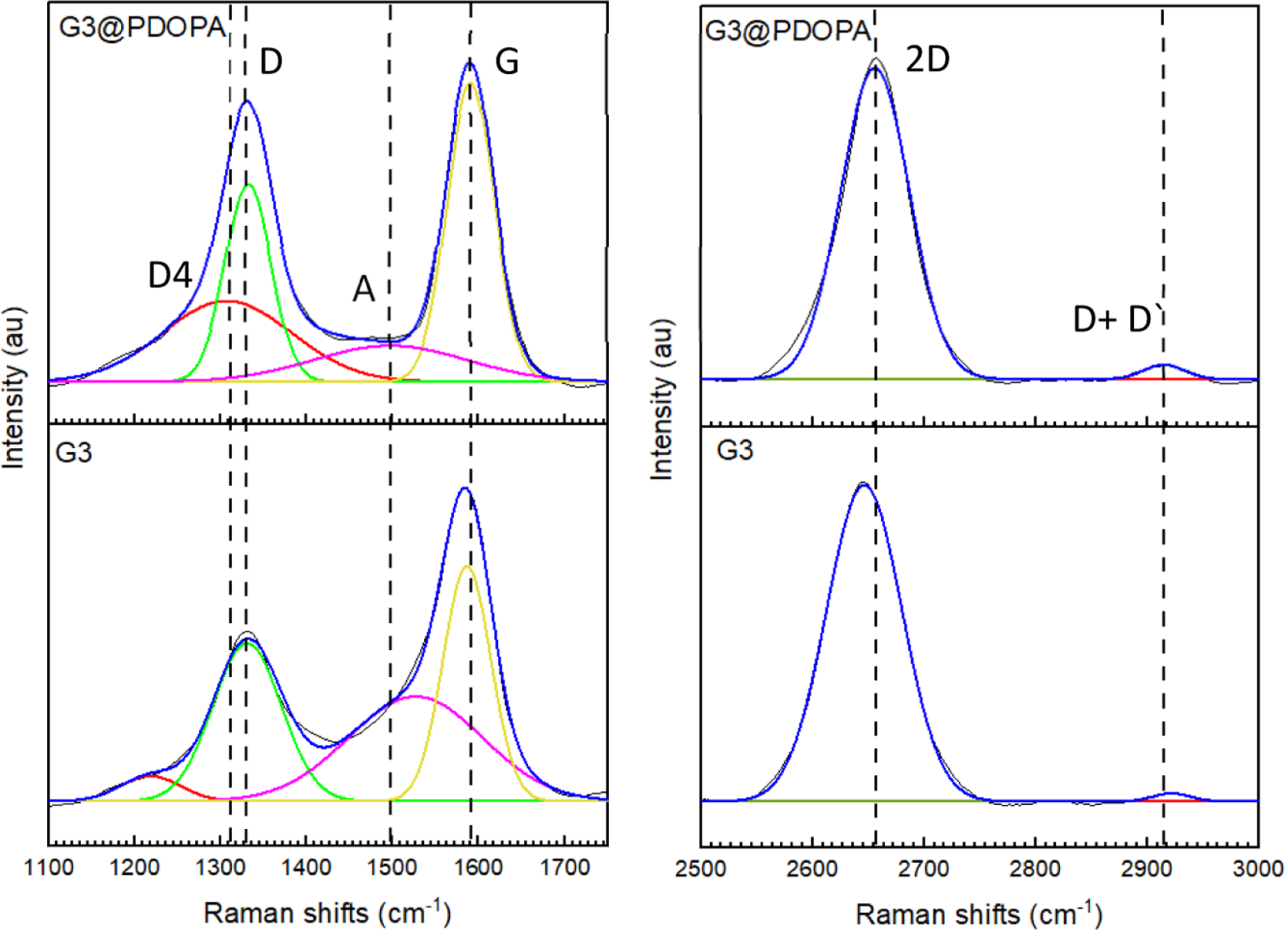
Original (blue) and deconvoluted (into separate bands; G—yellow, D—green, A—purple, and D4—red) Raman spectra of G3 graphene (bottom panel) and G3@PDOPA (top panel) at the critical regions.

As shown, pristine graphene exhibits high thermal stability, yielding residues of as high as 95 wt % up to 900 °C, which corresponds to the previous findings on the nature of oxygen functionalities.^79^ For PDOPA, the first weight loss (below 200 °C) in TGA could be connected with the desorption of water. Above 220 °C, PDOPA started to decompose, which was connected with the degradation of the catechol moiety and decompositional volatilization of the amine groups and decarboxylation.^54,80^ Thermal degradation of G3@PDOPA has been found to be qualitatively similar to that of pure PDOPA, although the degradation of the adsorbed polydopamine occurred at a higher temperature around 300–400 °C (Figure 5B), confirming the covalent nature of the PDOPA tethering. The TGA curve of G3@PDOPA revealed a steady weight loss between 300 and 900 °C, which can be attributed to the degradation of the PDOPA molecules with the concomitant aromatization in the macromolecules of the so-forming residue. Importantly, TGA runs revealed that there was ca. 23 wt % of PDOPA in G3@PDOPA, which in turn corroborated now well with the elemental analysis. In order to extend the analysis of surface physicochemistry upon functionalization, Raman spectroscopy was performed, revealing the characteristic features of the graphene derivatives (Figure 6).

The Stokes phonon energy shifts in graphene correspond to the three main peaks in the Raman spectrum: D-band (∼1330–1340 cm^–1^), G-band (∼1570–1590 cm^–1^), and the second-order band 2D at ∼2670 cm^–1^. According to Sadezky et al.’s deconvolution method, the spectra in the range of 1000–2000 cm^–1^ can be separated into the following bands: G, D, A, and D4.^81^ The G-band (∼1591 cm^–1^) corresponds to the ideal graphitic lattice, the D-band (∼1332 cm^–1^) to the disordered graphitic lattice (graphene edges, A_1g_ symmetry), A-band (∼1497 cm^–1^)—amorphous carbon, and D4-band (∼1308 cm^–1^)—disordered graphitic lattice (A_1g_ symmetry). The 2D-band (∼2656 cm^–1^) displays the highest intensity which is connected with the crystal size in the interlayer dimension.^82^ The other band is overtone D + D′ at 2913 cm^–1^. It should be noted that the D-band is typically qualitatively and quantitatively related to the non-sp^2^-C defects, that is, a number of sp^3^-C atoms. The G-band in the spectra of graphene samples revealed signals at 1586 cm^–1^ (pristine graphene) and at 1591 cm^–1^ (G3@PDOPA). The *I*_D_/*I*_G_ ratio could be taken as a measure of the sp^3^-carbon defect concentration. The crystallographic defects here can be assigned to the C atoms which underwent addition and subsequent polymerization. The *I*_D_/*I*_G_ ratio increased from ca. 0.54 (pristine graphene) to 0.88 (G3@PDOPA), indicating the conversion of sp^2^_-_ to sp^3^-C atoms in the basal plane of the functionalized graphene, confirming the earlier observations. The other Raman parameter of diagnostic relevance used for the characterization of graphene is the *I*_D_/*I*_2G_ ratio. The small *I*_D_/*I*_2G_ ratio is the evidence of high crystallographic order in the initial graphene structure. Therefore, after functionalization with PDOPA, the *I*_D_/*I*_2G_ ratio increased from ca. 0.80 (pristine graphene) to ca. 0.87 (G3@PDOPA). Nevertheless, not only intensity but also shifts of the D- and G-bands provide information on the functionalization of graphene. Therefore, while the positions of the D-band remained practically unchanged for all of graphene samples, the G-bands for G3@PDOPA were shifted to higher frequencies. To track the changes in the G-band positions, we have carried out a line-shape analysis to minimize the number of independent fitting parameters. After functionalization, the signals were shifted ca. 5 cm^–1^ to higher wavenumbers, indicating the consequence of electron transfer from the π-states of graphene upon trapping radicals generated at the stage of initiation of polymerization. Subsequently, the growth of the polymer chain proceeded from the graphene flake being an active sp^2^-carbon radical scavenger.^83^ Importantly, chemisorption of this first radical opens up the cascade of covalent bond formation,^84^ which eventually drives the process to the effective coating of graphene with PDOPA. This behavior has found clearly a reflection in the results we obtained from the physiochemical studies on G3@PDOPA.

### Rheological Characterization of Paints

**Figure 7:**
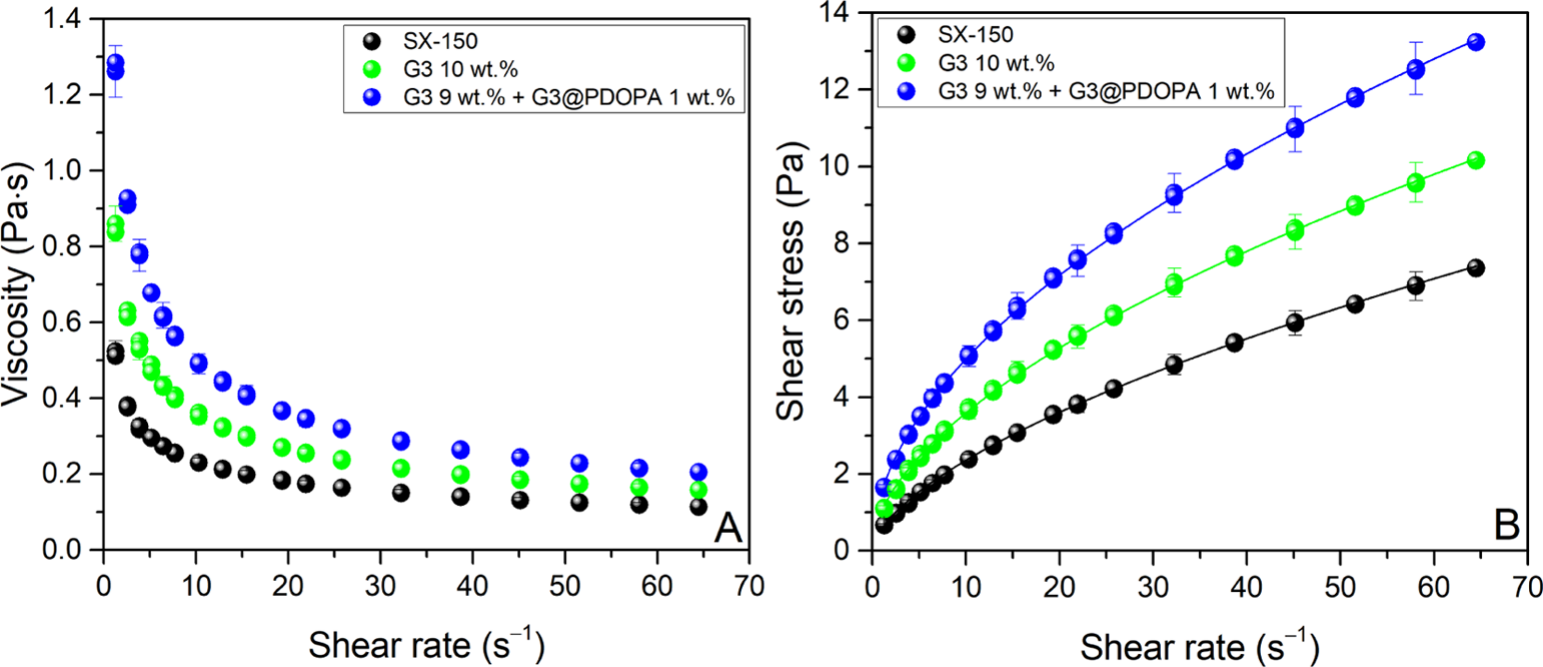
Rheological characteristics of paints: viscosity curves (A) and flow curves (B). Solid lines—values calculated from the Herschel–Bulkley model (*R*^2^ > 0.999).

The investigated paints at 40 °C are highly viscous, non-newtonian shear-thinning fluids, the viscosity of which declines with an increasing shear rate up to 83.7% for G3 9 wt % + G3@PDOPA 1 wt % (Figure 7). It is caused by shear-induced deagglomeration and orientation of particles in the flow direction, which reduce the internal fluid friction.^85^ In the application of paints, varnishes, and pigments, shear-thinning is a desirable phenomenon. High viscosity at low shear rates (i.e., gravitational conditions) helps maintain product consistency and prevents phase separation and running or streaking after application to a vertical surface, while low viscosity at medium/high shear rates during the processing and application of paints makes them easy to mix, pump, transport, and spread with brushes, rollers, and so forth.^86^ Furthermore, we observed very little difference between the shear rate ramp-up and ramp-down curves (no hysteresis loops); thus, the paints practically do not have time-dependent thixotropic properties. Such an almost immediate rebuilding of the fluid structure upon the application of stress/strain indicates that the paints will be devoid of sagging and droplet formation after application.^87^ In addition, the samples of G3 10 wt % and G3 9 wt % + G3@PDOPA 1 wt %, unlike the neat base SX-150, show the existence of a low yield stress τ_0_ of 0.10 and 0.12 Pa, respectively. The yielding behavior of paints is important in many areas, including the evaluation of shelf-life stability and sagging after application to the surface. Table 3 summarizes the rheological parameters of the samples determined based on experimental data and mathematical modeling with the Herschel–Bulkley model^88^
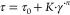
where γ̇ is the shear rate, τ is the shear stress, τ_0_ is the yield stress (experimentally determined), *K* is the consistency index, and *n* is the flow behavior index.

**Table 3:** Rheological Parameters of Paints Determined via Experimental Research and the Herschel–Bulkley Model

parameter, unit	SX-150	G3 10 wt %	G3 9 wt % + G3@PDOPA 1 wt %
τ_0_, Pa	0.00 ± 0.02	0.10 ± 0.02	0.12 ± 0.02
*K*, Pa s^n^	0.57 ± 0.018	0.94 ± 0.0092	1.42 ± 0.010
*n*, -	0.61 ± 0.0069	0.57 ± 0.0027	0.53 ± 0.0020

### Morphology and Electroconductivity of the Graphene Coatings

**Figure 8:**
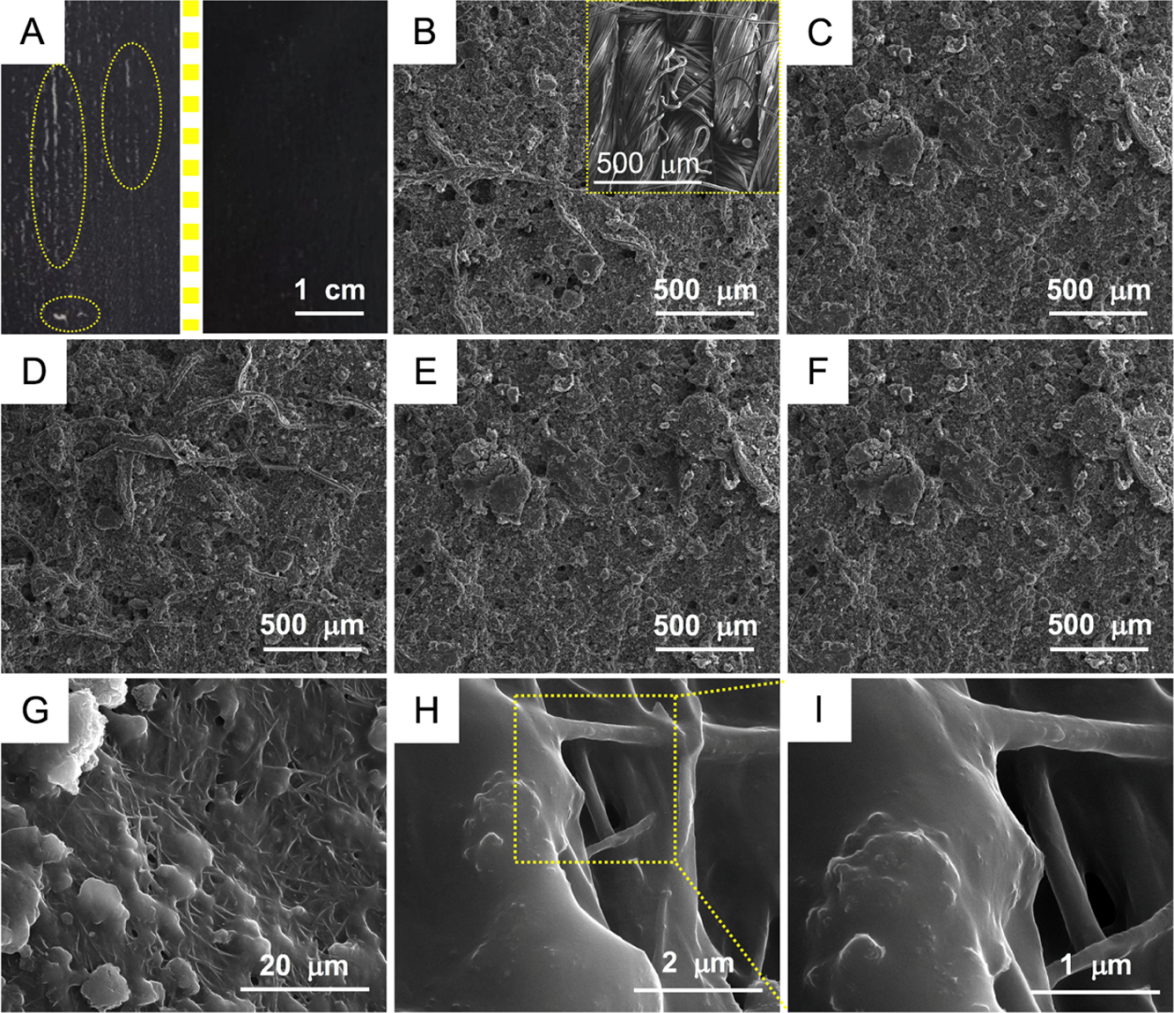
(A) Photograph of the conductive paths composed of pristine G3 (left) and G3@PDOPA (right); the indicated areas show discontinuous coverage by the paint. (B–F) SEM images of the conductive paths composed of 10 wt % G3, 10 wt % G3@PDOPA, 5 wt % G3 + 5 wt % G3@PDOPA, 8 wt % G3 + 2 wt % G3@PDOPA and 9 wt % G3 + 1 wt % G3@PDOPA; the inset in B shows the neat cotton textile. (G–I) Most electroconductive paths based on 9 wt % G3 + 1 wt % G3@PDOPA under increasing magnification, revealing the perfectly coated cotton filaments.

Having obtained the prospective characteristics, that is, the PDOPA capability of individualizing graphene flakes, we attempted to prepare a paint and consequently the functional, electroconductive textile coating. Figure 8A presents the photographs of the coating deposited on a cotton textile via a simple but controlled brush painting. Indeed, only the G3@PDOPA-based paint allowed for the homogeneous coating (right panel), opposite to the only-graphene counterpart (left panel). The analysis of the coatings was further conducted using SEM. Figure 8B–F shows the SEM micrographs of five coatings with different G3-to-G3@PDOPA ratios. Figure 8B represents coatings based on pristine graphene G3. Here, the paint did not cover the cotton textile uniformly as the larger (ca. 200 nm) spherical voids were clearly visible. Because of the poor dispersibility of graphene in water, it was very difficult to achieve a fine dispersion throughout the textile filaments as well as homogeneous distribution of graphene; indeed, in both cases, numerous and large agglomerates were visible. The incorporation of G3@PDOPA significantly improved the dispersibility of graphene, and the coatings were found to be more uniform (Figure 8C–F) with much smaller voids (<50 nm) than that for G3-based coatings. Importantly, the increasing magnifications (Figure 8G–I) of the (further confirmed) coating show an excellent homogeneity of the coverage found for the individual filaments.

**Figure 9:**
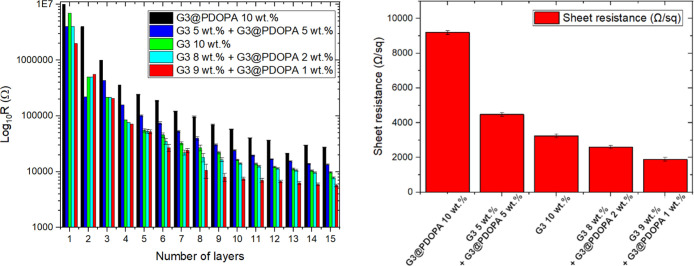
(Left) Electrical resistivity of graphene paints coated on cotton textiles with the total concentration of 10 wt %, and (right) sheet resistance after the deposition of the latest layer.

Sheet resistance measurement is the first-of-choice parameter describing the electrical properties of the coatings. It is also a method which immediately informs whether further deposition of new layers of paint would bring any significant changes in the electrical conductivity. As presented in Figure 9 (left), there is a clear decreasing dependence of resistance with the increasing number of layers. For deposited paints composed of 10 wt % pristine graphene, the electrical resistivity decreases from 7 MΩ to 9.8 kΩ, while in the case of 10 wt % functionalized graphene G3@PDOPA, it decreases from 10 MΩ to 27.6 kΩ.

Deterioration of the electrical properties of G3@PDOPA was caused by a high content of PDOPA ∼23 wt %. However, after the deposition of the paints containing a mixture of pristine graphene and G3@PDOPA, the electrical properties were synergistically augmented. We studied lower (1–5 wt %) weight fractions of G3@PDOPA and found that the lowest resistance could be achieved for the composite containing 1 wt % of G3@PDOPA (per total conductive phase content). The electrical resistance after the deposition of 15 layers (final thickness ca. 100 μm; Figure 9 (right)) is in the following order: 13.44 kΩ (5 wt % G3 and 5 wt % G3@PDOPA), 7.8 kΩ (8 wt % G3 and 2 wt % G3@PDOPA), and 5.7 kΩ (9 wt % G3 and 1 wt % G3@PDOPA). Recalculating the latter value to the sheet resistance yields 1.9 Ω sq^–1^, which is slightly lower^89^ or an order of magnitude lower^90^ than that reported previously for the green solvent-based inks. Importantly, from the mechanical properties point of view (Supporting Information, Figure S7), the resistance for the most conductive and graphene-only coating upon 100 bending cycles from 180 to 0° (a sharp edge) increased to 8 and 10 Ω sq^–1^, respectively. At the same time, the most perspective cotton coating G3 9 wt % + G3@PDOPA 1 wt % emerged as more resistant to washing and strain than G3 10 wt %, with an increase in Δ*R*/*R*_0_ as low as 7% after five cycles of washing/drying and a 90% increase upon 10% elongation, respectively (although with the similar characteristics upon 500 g loading/unloading in 50 cycles).

In summary, although G3@PDOPA is a much worse nanofiller than pristine graphene because of the high PDOPA content, it has the potential to be used as a supramolecular dispersing agent. Similar behavior was observed for oxidized carbon nanotubes (CNTs)^91^ or nitrogen-doped carbon nanobubbles,^92^ stabilizing the dispersions of long nanotubes or in the case of dispersions of short versus long multiwalled CNTs.^59^ Here again, the formation of novel, water-dispersible hybrids resulted from the π–π stacking interactions between graphene and the surfactant-like G3@PDOPA individuals bearing negatively charged backbones pointed toward water.

## Conclusions

We demonstrated the water-based hence green approach to the covalently functionalized graphene via the bioinspired “growth-from” polymerization of l-DOPA. Our method allows for a prompt and simple production of excellent and time-stable aqueous graphene dispersions—at the same time opening up opportunities for the sustainable use of the green surface compatibilizers like PDOPA. The cross-verified method emerges as an attractive alternative to the most frequently used ones, that is, those exploring structurally deeply altered graphene relatives—GO and rGO. It should be emphasized that it is also an effective way to modify the hydrophilicity and biocompatibility of graphene toward the awaiting biomedical applications. Importantly, the uniform coating of graphene with PDOPA was achieved via thermally driven polymerization of LDOPA. The PDOPA content in the hybrid graphene/PDOPA solid reached 23 wt %, while the coating rich in hydroxyl, carboxyl, and amine moieties guaranteed excellent wettability with water. Taking the results toward upscaling, we have demonstrated that the graphene@PDOPA nanohybrid acts as a dispersing agent for the pristine graphene flakes, allowing to form long-lasting acrylic dispersions. As a consequence, flexible and printable/paintable coatings of significantly lowered electrical resistance up to 1.9 kΩ sq^–1^ could be straightforwardly manufactured, minimizing the need for graphene functionalization which, in turn, lowers the cost and the environmental impact. These value-added characteristics make graphene@PDOPA hybrids suitable for textronic sensors, charge dissipation, or EMI shielding. Importantly, the modification appears as directly extendable to other sp^2^-carbon allotropes such as CNTs, fullerenes, nano-onions, nanohorns, or quantum dots.

## Supporting Information Available

DLS profiles, zeta-potential distribution, ^1^H NMR, and ESI-MS spectra of G3, PDOPA, and G3@PDOPA as well as changes of electrical properties of the coatings upon mechanical testing and washing (PDF)
