# Supplementary material for: Biomimetically Inspired Highly Homogeneous Hydrophilization of Graphene with Poly(l-DOPA): Toward Electroconductive Coatings from Water-Processable Paints
Source: ACS Sustain Chem Eng. 2022 May 10;10(20):6596–608. doi: 10.1021/acssuschemeng.2c00226 (PMC9131455; doi:10.1021/acssuschemeng.2c00226)
Supplement: Supplementary file 1 — sc2c00226_si_001.pdf [file sc2c00226_si_001.pdf]

Supporting Information

for

Biomimetically-inspired highly homogenous hydrophilization of graphene with poly(L-dopa): toward electroconductive coatings from water-processable paints

Anna Kuziel[1,2], Grzegorz Dzido[3], Rafał G. Jędrysiak[1], Anna Kolanowska[1], Bertrand Jóźwiak[1,3], Juliette Beunat[2,4], Emil Korczeniewski[5], Monika Zięba[5], Artur P. Terzyk[5], Noorhana Yahya[6,7], Vijay Kumar Thakur[2,8,9], Krzysztof K. Koziol[2]*, Sławomir Boncel[1]*

[1] Department of Organic Chemistry, Bioorganic Chemistry and Biotechnology, Silesian University of Technology, Krzywoustego 4, 44-100 Gliwice, Poland

[2] Enhanced Composites and Structures Centre, School of Aerospace, Transport and Manufacturing, Cranfield University, Cranfield, Bedfordshire MK43 0AL, United Kingdom

[3] Department of Chemical Engineering and Process Design, Silesian University of Technology, Strzody 7, 44-100 Gliwice, Poland

[4] Cambridge Graphene Centre, Engineering Department, University of Cambridge, 9 JJ Thomson Avenue, Cambridge, United Kingdom

[5] Faculty of Chemistry, Physicochemistry of Carbon Materials Research Group, Nicolaus Copernicus University in Toruń, Gagarin Street 7, 87-100 Toruń, Poland

[6] Department of Fundamental and Applied Sciences, Universiti Teknologi Petronas, 32610 Seri Iskandar, Perak Darul Ridzuan, Malaysia

[7] Spin Eight Nanotechnologies Sdn. Bhd. 28 Persiaran Jelapang Maju 7, Kawasan Perindustrian Ringan Jelapang Maju 30020 Ipoh, Malaysia

[8] Biorefining and Advanced Materials Research Center, SRUC, EH9 3JG Edinburgh, UK

[9] School of Engineering, University of Petroleum & Energy Studies (UPES), Dehradun

248007, India

Supporting Information is presented on seven (7) pages (S1-S7), and contains 7 figures (Fig. S1-S7) and 1 table (Table S1).

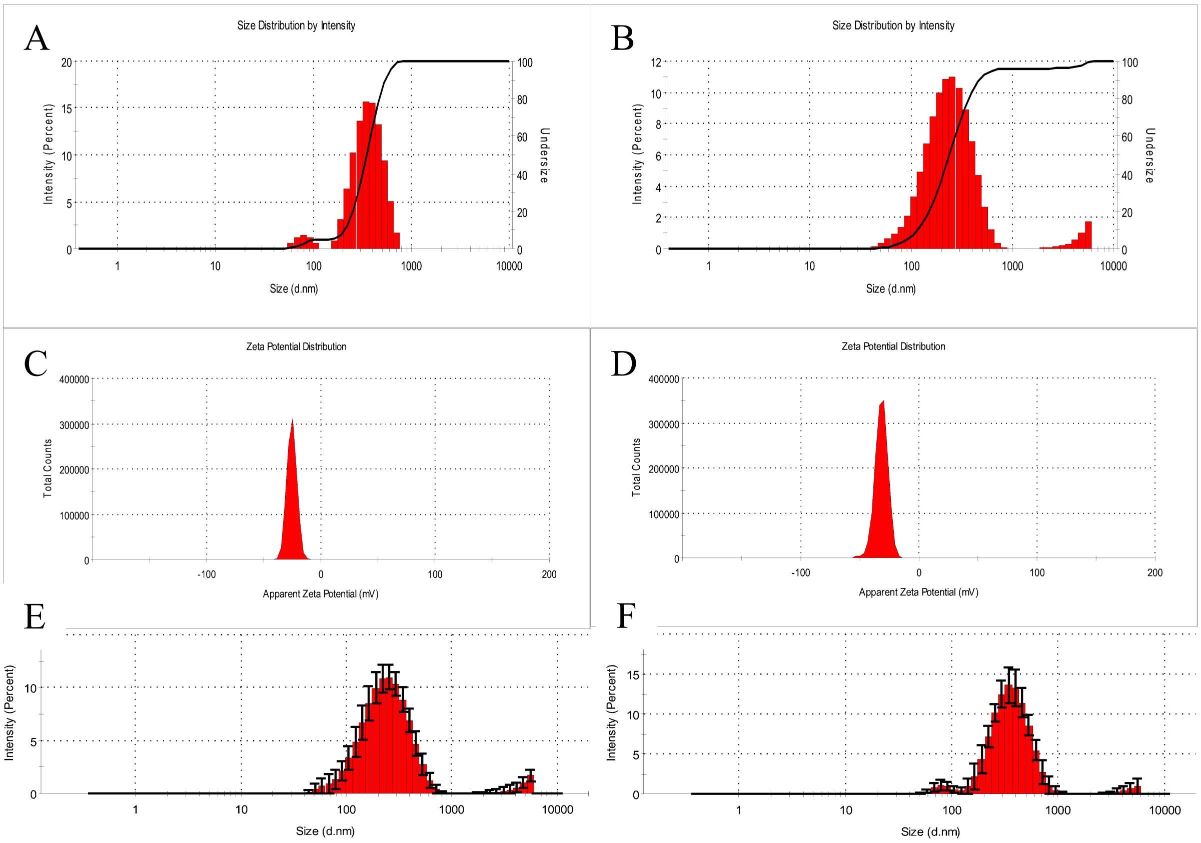

Fig. S1 Clarified DLS profiles of PDOPA (**A**) and G3@PDOPA (**B**) with the corresponding figures containing error bars (**E**) and (**F**); zeta potential of PDOPA (**C**) and G3@PDOPA (**D**).

Table S1

Sample	T, °C	z-Average* d, nm	Polydispersity index (PDI)	Diameter d, nm (%)		
PDOPA	25.0	**222.5**<br>SD = 2.4	0.350	**253.2**<br>95.9%	**4687**<br>4.1%	0.000<br>0.0%
G3@PDOPA	25.0	**341.8**<br>SD = 18.8	0.322	**376.6**<br>93.6%	**85.84**<br>4.1%	**4789**<br>2.2%

* The z-average values given in the table are the arithmetic mean of five independent measurement series; SD = standard deviation

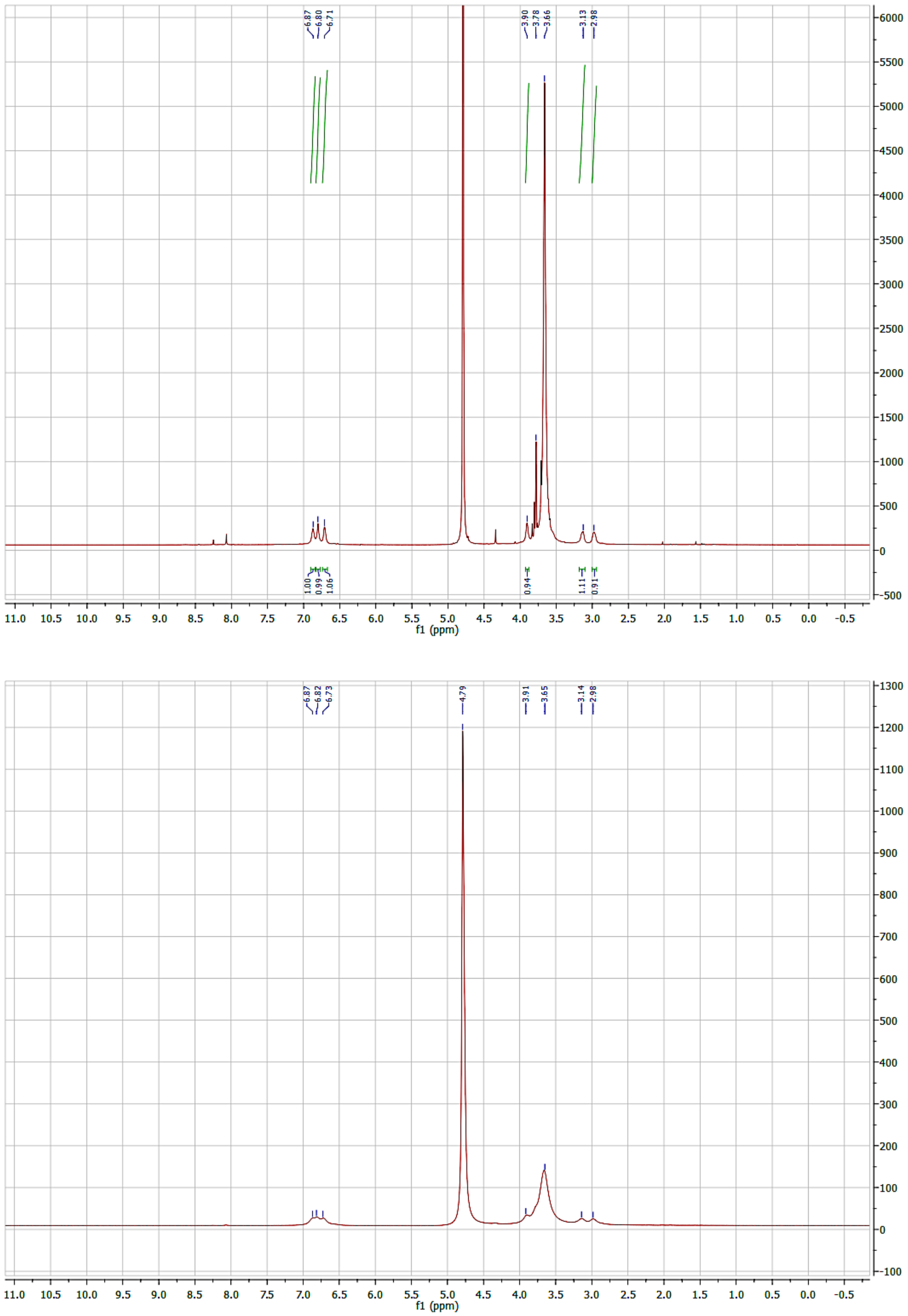

Fig. S2 ^{1}H NMR spectra of PDOPA (*top*) and G3@PDOPA (*bottom*) in D_2O

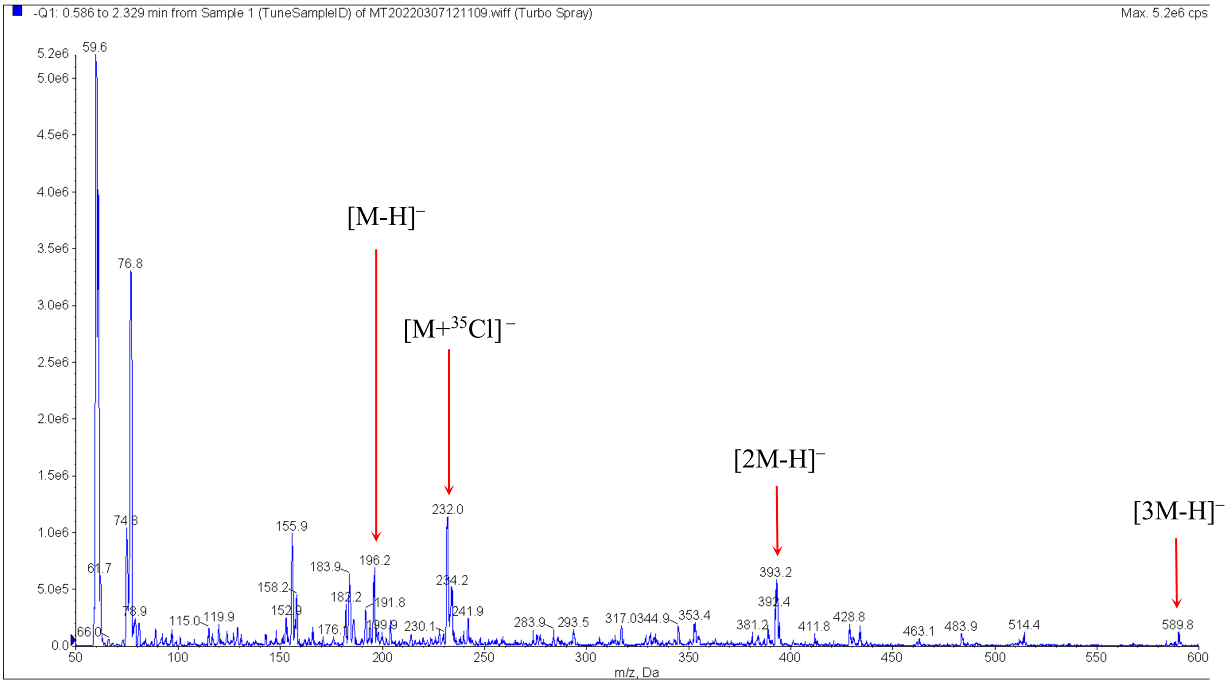

Fig. S3 ESI-MS spectrum of PDOPA in the negative ionization mode ($M=M_{LDOPA}$ = 197.07)

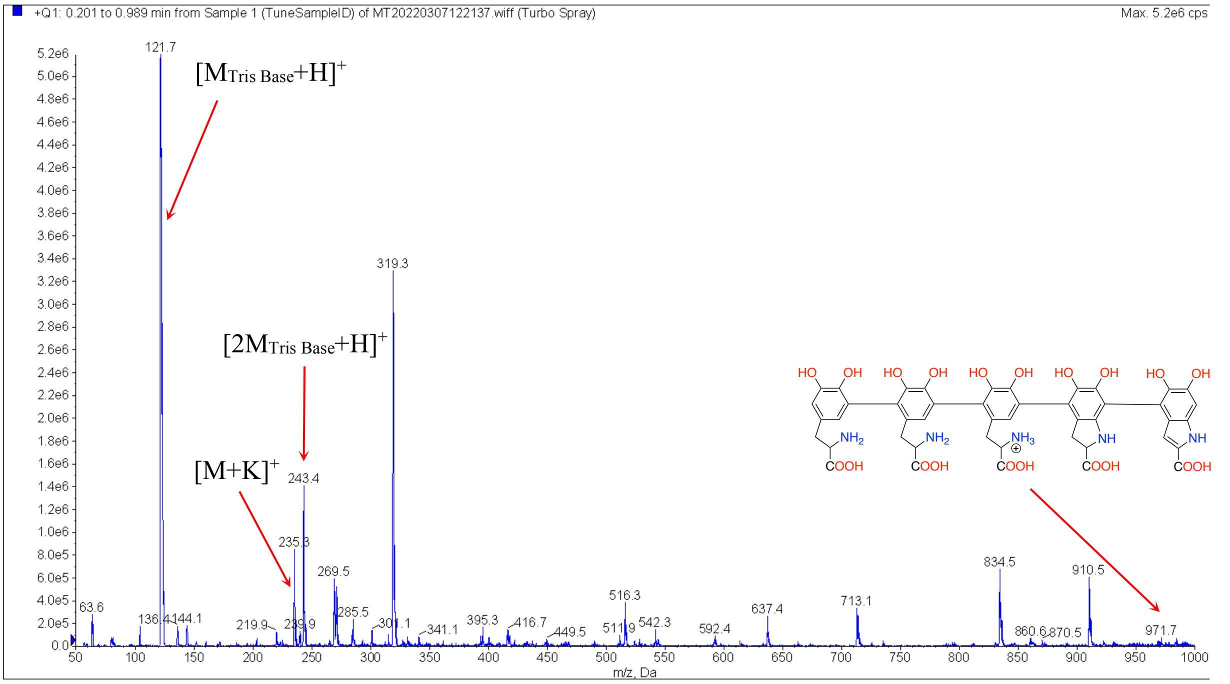

Fig. S4 ESI-MS spectrum of PDOPA in the positive ionization mode

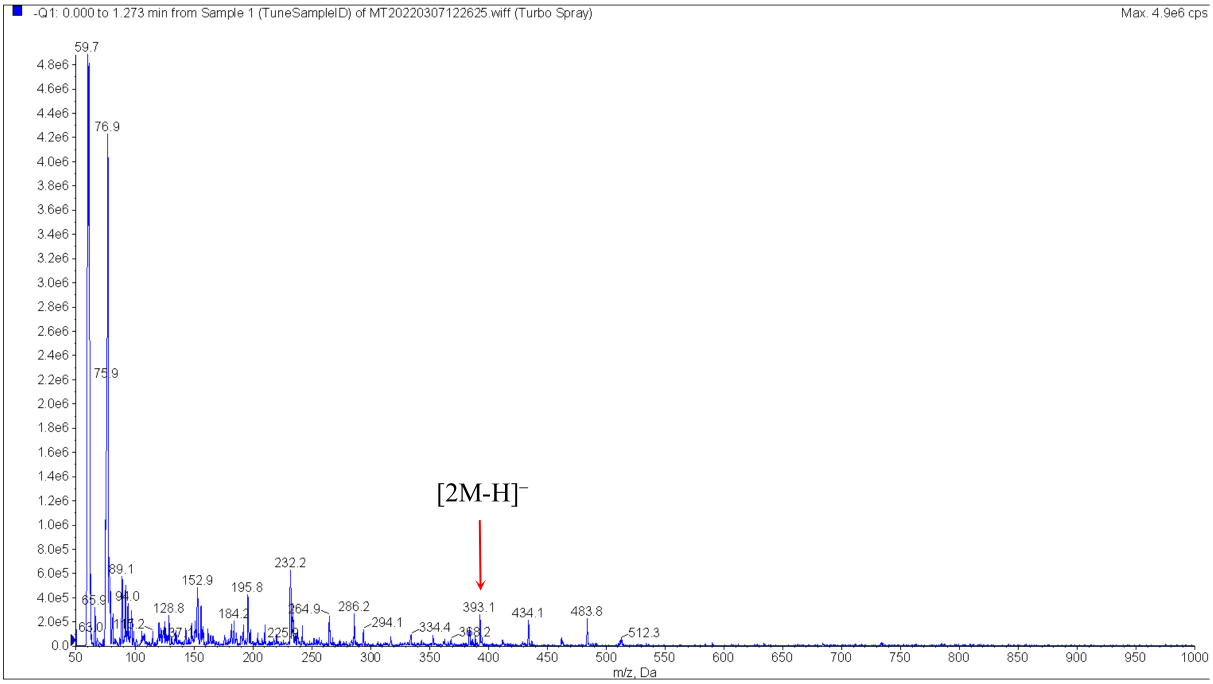

Fig. S5 ESI-MS spectrum of G3@PDOPA in the negative ionization mode

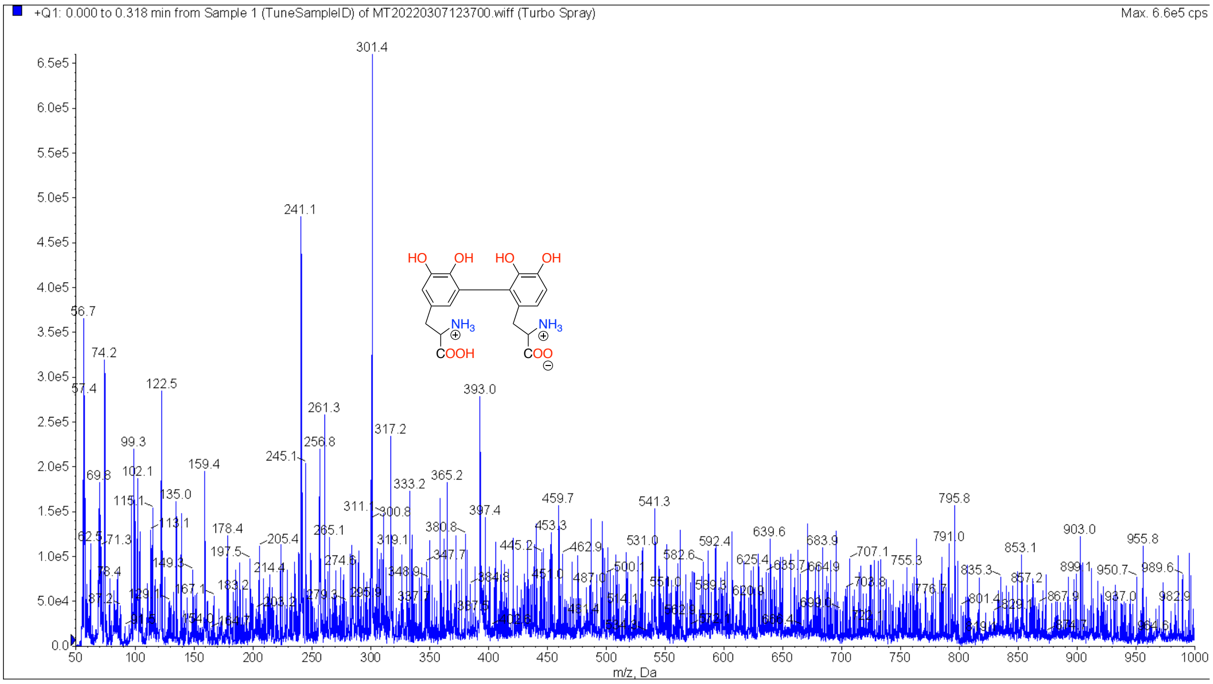

Fig. S6 ESI-MS spectrum of G3@PDOPA in the positive ionization mode

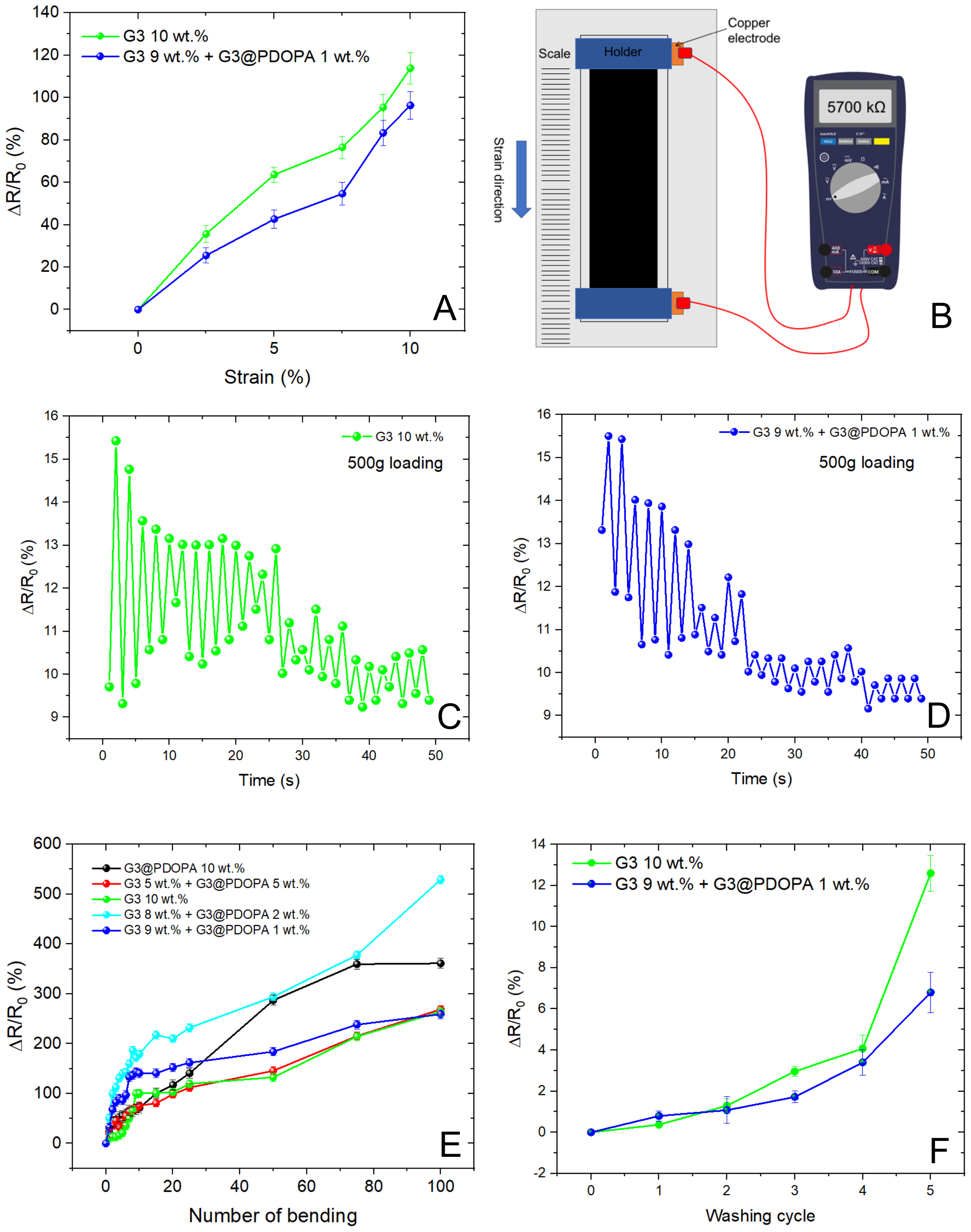

Fig. S7 (**A**) The changes upon electrical resistance upon: controlled strain (**A**) and stretching as determined by repeated loading/unloading cycles using a 500 g-weight on the e-textile (coated textile) strips, and the resistance value monitored using a multimeter for G3 10 wt.% with the experimental setup (**C**) and G3 9 wt.% + G3@PDOPA 1 wt.% (**D**), and the corresponding custom designed experimental setup (**B**). Electrical resistance upon: 100 bending cycles from 180 to 0 degrees (a sharp edge) (**E**) and washing (**F**) – vigorous agitation in 0.2 wt.% SDS aqueous solution (300 rpm, 2 h for the first four cycles. Then, the washing time was increased to 12 h in the fifth cycle. All of the tests were performed for the three individual sets of samples.
